# Crew resource management and threat and error management improve team communication in endoscopy: a prospective study

**DOI:** 10.1038/s41598-025-21475-8

**Published:** 2025-11-03

**Authors:** Dominik Schweikart, Anna Melzer, Niklas Sturm, Benjamin Mayer, Martin Müller, Martin Wagner, Thomas Seufferlein, Matthias Baur, Dominique Walter, Benjamin M. Walter

**Affiliations:** 1 Present Address: Endoscopy Research Unit, University Hospital Ulm, Albert-Einstein-Allee 23, 89081 Ulm, Germany; 2 Clinic for Internal Medicine I, Department of Gastroenterology, University Hospital Ulm, Ulm, Germany; 3 Institute of Epidemiology and Medical Biometry, Ulm University, Ulm, Germany; 4 CRM Trainer, Commercial Airline Pilot, Frankfurt, Germany

**Keywords:** Patient safety, Teamwork, Team communication, Learning from aviation, Colonoscopy, Oesophagogastroscopy

## Abstract

In gastrointestinal endoscopy, the ability to perform increasingly complex procedures has significantly heightened the demand for effective team cooperation. Crew Resource Management (CRM) and Threat and Error Management (TEM), proven strategies from civil aviation, have the potential to enhance team communication, workflow and patient outcomes in complex medical settings such as interventional endoscopy and therefore were implemented into endoscopy unit in this study. Together with qualified CRM trainers, individually tailored CRM and TEM elements such as communication guidelines, checklists and TEM-based dialogic team-time-outs were developed with the employees and integrated into endoscopic workflow at a high-volume interventional endoscopy center. Employee surveys on the effects were performed before and after the implementation. Implementation of CRM and TEM elements into endoscopy with civil aviation experts improved team communication, workflow and teamwork in endoscopy. Our study found a significantly reduced rate of misunderstandings caused by ambiguous communication during endoscopic interventions (*p* = 0.034), a significantly clearer defined distribution of tasks during the procedure (*p* = 0.020) and a significantly improved physicians’ information transfer to the nursing assistants before the procedure (*p* = 0.047). More than 80% of all employees perceived that patient safety had improved after the intervention.

**Supplementary Information:**

The online version contains supplementary material available at 10.1038/s41598-025-21475-8.

## Introduction

Gastrointestinal endoscopy enables rapid and precise diagnosis of many gastrointestinal diseases. In recent decades, the scope of therapeutic endoscopy has expanded significantly, resulting in increasingly complex interventions. This complexity has led to a substantial rise in the physical and psychological demands placed on both the endoscopy team and individual endoscopists. Despite these challenges, the primary quality objective of medical care is to guarantee patient safety, which requires optimal team performance within the field of endoscopy. Drawing inspiration from civil aviation, specific approaches such as Crew Resource Management (CRM) and Threat and Error Management (TEM) have shown promise in meeting the demands of complex endoscopic workflows. Aviation’s status as the safest mode of transportation today is largely due to decades of dedicated efforts to address human factors.

CRM originated in 1979 from a National Aeronautics and Space Administration (NASA) workshop that identified human errors in leadership, interpersonal communication and decision-making and as the root cause of many air traffic accidents^1,2^. CRM aims to optimize the use of all available resources and non-technical skills such as communication, teamwork and decision-making, thereby improving the security of work processes^1,3–5^. CRM can manage errors in high-risk organizations with error countermeasures through three lines of defense: Firstly “avoidance of errors”^1^, secondly capturing “incipient errors before they are committed”^1^, and thirdly mitigating the consequences of unavoidable errors^1,2^. TEM is a part of these countermeasures, as the proactive identification of threats and possible sources of error in work processes as well as the prevention and management of the resulting potential hazards are its basic principles^1,6,7^. An important component of CRM is assertiveness, which is characterized by the ability to openly express opinions, address problems, demand and give help when necessary and propose solutions within the team, regardless of hierarchy, and while respecting the views of other team members^8,9^. The priority is to ensure that the team is aware of the information provided so that a decision can be made within the team to resolve the situation in the best possible way and avoid possible subsequent events and is therefore essential for safe and effective team performance. Another possible inspiration of aviation is the principle of closed loop communication. A team member sends a message to the recipient, who clearly confirms receipt of the message^10^. The sender then makes sure that the message has arrived and was interpreted as intended and the “loop” is complete. It was shown that closed loop communication can have a positive influence on team communication, efficiency and dynamics in healthcare settings^11,12^.

Acknowledging the critical role that human factors—such as teamwork and communication—play in preventing adverse events during endoscopic procedures is a crucial step towards enhancing optimal workflow. The focus of implementing CRM elements into endoscopy should aim to improve team dynamics and reduce the likelihood of errors, ultimately leading to better patient outcomes^13^. The WHO Surgical Safety Checklist of 2009 showed that elements of CRM and TEM can have significant effects on mortality, morbidity and other important clinical outcomes^14,15^. Hefner et al. observed a general improvement in patient safety in the entire medical center and a significant improvement in teamwork, patient safety, communication and feedback culture following a CRM-based training, e.g. on checklists throughout the hospital^16^. Previous studies investigated the beneficial introduction of a generalized safety checklist in endoscopy^17–20^. Dubois et al. found that a safety checklist improved patient identity verification rates in the endoscopy unit but couldn’t find significant effects on teamwork and communication^17^. Kherad et al. showed that team communication and team satisfaction in the endoscopy unit profited after the implementation of a self-developed checklist. They also hypothesized that in addition to the safety checklist, communication guidelines could also play a role in improving team communication, but this was not investigated in the study^18^. It was also shown that safety checklists can improve patient verification rates in endoscopy and reduce response time to emergency endoscopy^19,21^. Analogously to the WHO Surgical Safety Checklist, the ESGE/ESGENA GI Endoscopy Safety Checklist of 2022 was introduced as a standardized checklist available for all endoscopy units^22^. It included e.g. a team time-out and pre- and post-interventional safety measures. Although such a standardized checklist is an important guide for endoscopy units, we believe that given the challenges of modern endoscopy, it may be an alternative to take an individualized approach to endoscopy safety, while still integrating the necessary expert knowledge. ESGE and ESGENA also mentioned that the adaption of the checklist on local requirements is an option for endoscopy units^22^. Therefore, in our study we want to investigate whether an individual approach with CRM and TEM measures developed in direct cooperation with experts from civil aviation for the respective endoscopy unit can also improve team communication and teamwork. These included a standardized communication guideline as an addition to closed loop communication, a safety checklist and a TEM-based dialogic team-time-out in endoscopy. To the best of our knowledge, our study is the first to investigate the possibility of individually implementing tailored CRM and TEM measures for the respective endoscopy unit in direct cooperation with experts from civil aviation.

## Methods

**Fig. 1:**
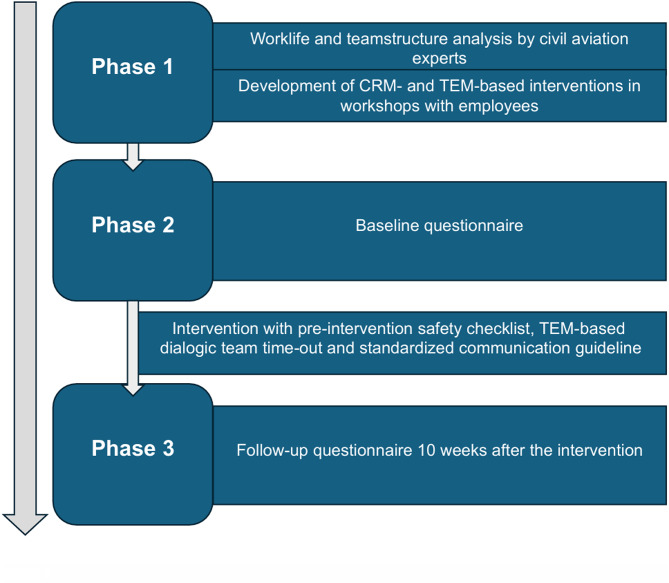
Study procedure: the study was conducted in three phases.

A prospective study was conducted at the interventional endoscopy center at University Hospital Ulm, Germany, to assess the impact of CRM-based measures on team communication, worklife and teamwork of the endoscopic unit (Fig. 1). Firstly, a specialized survey was conducted within the endoscopy unit which analysed different factors of working life. Based on these results, workshops specifically adapted to the resulting demands were conducted for employees by two qualified CRM-trainers and commercial airline pilots from civil aviation. Within these workshops, basics of CRM, TEM and assertiveness were elaborated and elements from CRM and TEM adapted to endoscopy were developed over the period of three full days. These included a pre-intervention safety checklist, a TEM-based dialogic team-time-out and a standardized communication guideline. The safety checklist contained the most important information about the patient like e.g. patient identification or laboratory values. The TEM-based dialogic team-time-out also involved both, physicians and nursing assistants, in the time-out process before the procedure and additionally included strategies for recognition and mitigation of potential errors and adverse events. The standardized communication guideline (Supplementary material A) defined the communication between physicians and nursing assistants regarding various interventional devices such as e.g. forceps or clips with the aim of preventing ambiguous communication and resulting misunderstandings. In addition to closed loop communication the aim here was to ensure that the same processes carried out during interventions are named in the same way by all team members. These elements were introduced to employees with presentations of a designated implementation leader of the endoscopy team over the period of two days and printed on cards that were displayed in each operating room. Following their introduction, the measures were implemented in the endoscopic workflow and have been used consistently ever since.

The effects of these measures were examined by employees of the endoscopy unit voluntarily by completing an anonymous questionnaire (Supplementary material B) adapted to the professional group before (*n* = 25) and 10 weeks after (*n* = 26) the measures were introduced. The questionnaire was based on standard CRM questions from aviation as well as questions particularly suitable for employee surveys, which were specifically adapted to the endoscopy department^23^. For the survey a 5 item Likert scale was used. The second survey was expanded by 10 questions on the introduction of the CRM measures. In accordance with Declaration of Helsinki for this study, ethical approval was obtained by the local ethic committee. Informed consent was obtained from all participants.

The primary objective was to analyse differences in perception of team communication after the intervention.

Secondary objectives were the evaluation of alterations in workflow, teamwork, working life, feedback culture, stress and behaviour in emergency situations in the endoscopy unit before and after the CRM implementation. Furthermore, the overall perception of the implementation of the CRM and TEM measures was to be examined with the additional questions. Another secondary objective was the assessment of the additional time required to complete the checklist and the TEM-based dialogic team-time-out to investigate possible delays in workflow and the influence of increasing routine on the time needed. For this purpose, procedure time was measured on 10 consecutive days during regular endoscopy sessions.

With regard to sample size, no power calculation was performed due to the limited number of employees in the endoscopy unit. The collected data were analysed using SPSS (version 29.0.1.0 172). A numerical value was assigned to each answer option on the Likert scale (5.00 = I fully agree, 4.00 = I tend to agree, 3.00 = partly/partly agree, 2.00 = I tend to disagree, 1.00 = I strongly disagree). Descriptive statistics with mean, standard deviation, median, interquartile range (IQR), minimum and maximum were prepared for the analysis. The Mann-Whitney-U test was used as a non-parametric hypothesis test for the comparison between the baseline and the follow-up survey. A Fisher-Freeman-Halton exact test was used for dichotomous responses as gender and professional groups. A t-test was used as a hypothesis test to analyse the time differences of performing the checklist before the intervention. P-values < 0.05 were assumed to be significant. For the hypothesis tests, asymptotic significance was used for a sample size > 30 and exact significance for a sample size < 30. If data was missing for individual questions, the missing data was not included in the corresponding evaluation of the specific question.

## Results

The first survey (baseline) was completed by 25 staff members and the follow-up survey after the introduction of CRM measures (safety checklist, TEM-based dialogic team-time-out and standardized communication guideline) by 26 staff members (Table 1). For physicians, the response rate for the questionnaire was 90% at baseline and follow-up, for nursing assistants 89% at baseline and 94% at follow-up. Further study population characteristics can be found in Table 1.

**Table 1:** Study population characteristics.

	Baseline survey	Follow-Up survey
Sex		
Male n (%)	10 (40.00)	11 (42.31)
Female n (%)	15 (60.00)	15 (57.69)
Age range		
21–35 n (%)	6 (24.00)	7 (26.92)
36–50 n (%)	10 (40.00)	10 (38.46)
51–65 n (%)	9 (36.00)	8 (30.77)
> 65 n (%)	0 (0.00)	1 (3.85)
Profession		
Physicians n (%)	9 (36.00)	9 (34.62)
Nursing assistants n (%)	16 (64.00)	17 (65.38)

**Fig. 2:**
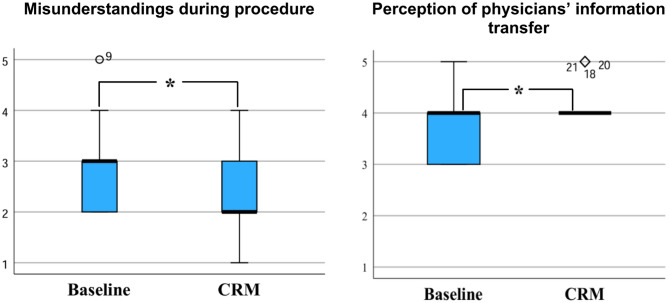
Team communication before and after implementation of safety checklist, team time-out and communication guideline; Less misunderstandings during the procedures and improved information transfer from the were shown after the intervention; 5 = I fully agree, 4 = I tend to agree, 3 = partly/partly agree, 2 = I tend to disagree, 1 = I strongly disagree; * *p* < 0.05.

When looking at communication within the team (Fig. 2), there was a significant reduction in perception of misunderstandings during the interventions (*p* = 0.047) after introduction of the CRM measures. In addition, our results showed increased medians regarding the discussion of conflicts (median (IQR): 3.00 (3.00–4.00) baseline vs. 4.00 (3.00–4.00) CRM, *p* = 0.269) and better availability of information (3.00 (3.00–4.00) baseline vs. 4.00 (3.00–4.00) CRM, *p* = 0.134).

Among physicians, there were increased medians for a more open communication (non-significantly) with other physicians about concerns during the procedure (4.00 (3.00-4.50) baseline vs. 5.00 (3.50-5.00) CRM, *p* = 0.258) and requests for support (4.00 (2.00–5.00) baseline vs. 5.00 (4.00–5.00) CRM, *p* = 0.297). Both professional groups showed improved communication with nursing assistants and physicians. The nursing assistants perceived the physicians’ information transfer about the patients before the procedure as significantly improved (*p* = 0.020).

No significant changes were found yet regarding the feedback culture and the level of stress. In both surveys, satisfaction with general working life in endoscopy was high and did not significantly change after the introduction of the CRM measures.

**Fig. 3:**
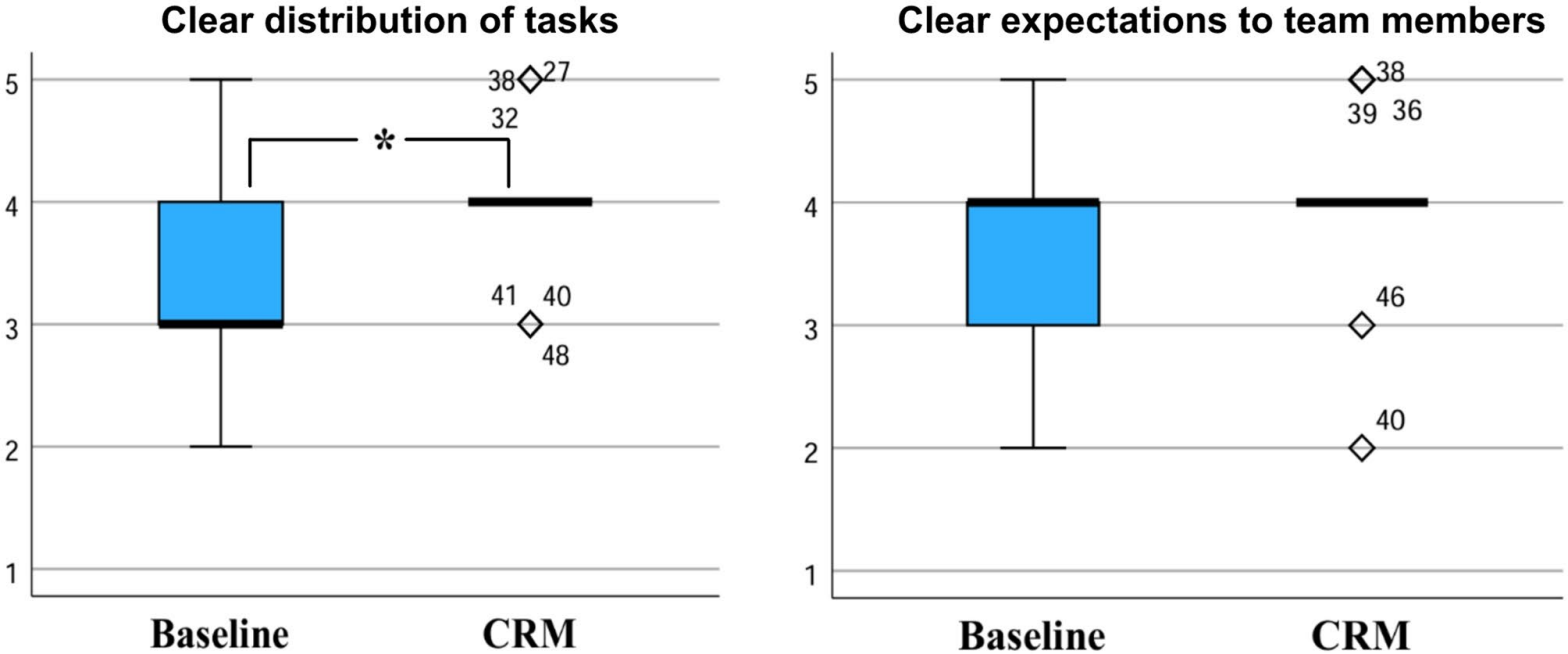
Workflow before and after implementation of safety checklist, team time-out and communication guideline: A clearer distribution of tasks as wells as clearer expectations to team members were perceived after the intervention; 5 = I fully agree, 4 = I tend to agree, 3 = partly/partly agree, 2 = I tend to disagree, 1 = I strongly disagree; * *p* < 0.05.

The workflow in the endoscopy department (Fig. 3) showed a significantly clearer defined distribution of tasks during the entire procedure after the introduction of the CRM measures (4.00 (3.00–4.00) baseline vs. 4.00 (4.00–4.00) CRM, *p* = 0.034). In the subgroup of physicians, the same question also showed a significance (*p* = 0.040). There was also a higher median for coordination during the interventions (4.00 (3.00–4.00) baseline vs. 4.00 (4.00–5.00) CRM, *p* = 0.120) and a non-significant tendency that it became clearer what could be expected from which team member and when (4.00 (3.00–4.00) baseline vs. 4.00 (4.00–4.00) CRM, *p* = 0.052) after the intervention. In the case of physicians, a higher median was found for paying more attention to ensure that all team members had the same level of knowledge at the beginning of the procedure (3.00 (3.00-3.50) baseline vs. 4.00 (3.00-4.50) CRM, *p* = 0.190).

After the introduction of the measures more employees agreed that all resources for the prevention of complications were available (4.00 (4.00-4.50) baseline vs. 4.00 (4.00–5.00) CRM, *p* = 0.390) and that these were used more effectively (4.00 (3.00–4.00) baseline vs. 4.00 (3.75-4.00) CRM, *p* = 0.371).

When asked about the measures introduced (Table 2), 76.9% of employees stated that the measures would hardly or not at all unnecessarily delay the endoscopic procedure (2.00 (1.00-2.25)). More than 80% of employees fully agreed or tended to agree that the safety checklist (4.00 (4.00–5.00)), the TEM-based dialogic team-time-out (4.00 (4.00–5.00)) and the communication guideline (4.00 (4.00–5.00)) had improved team communication and teamwork. Similarly, more than 80% of employees agreed completely or tended to agree that the safety checklist (4.00 (4.00–5.00)), the TEM-based dialogic team-time-out (4.00 (4.00–5.00)) and the communication guideline (4.00 (4.00–5.00)) had improved patient safety. 88.5% of employees would support the use of a safety checklist for an endoscopic examination performed on themselves (5.00 (4.00–5.00).

**Table 2:** Staff perception on CRM- and TEM-based measures.

	I agree (I fully agree/ I tend to agree) *n* (%)	Partly/Partly agree *n* (%)	I disagree (I tend to disagree, I strongly disagree) *n* (%)
In my opinion, the introduction of the safety checklist and the team time-out has unnecessarily delayed the examination process	5 (19.23)	1 (3.85)	20 (76.92)
In my opinion, the safety checklist has improved team communication and teamwork in endoscopy	21 (80.76)	3 (11.54)	2 (7.69)
In my opinion, the team-time- out has improved team communication and teamwork in endoscopy	22 (84.61)	2 (7.69)	2 (7.69)
In my opinion, the standardized communication guideline has improved team communication and teamwork in endoscopy	22 (84.61)	4 (15.38)	0 (0.00)
In my opinion, the safety checklist has improved patient safety	22 (84.61)	2 (7.69)	2 (7.69)
In my opinion, the team-time- out has improved patient safety	21 (80.76)	4 (15.38)	1 (3.85)
In my opinion, the standardized communication guideline has improved patient safety	21 (80.76)	4 (15.38)	1 (3.85)
I would prefer the use of a safety checklist for an endoscopic examination performed on me	23 (88.46)	3 (11.54)	0 (0.00)

**Fig. 4:**
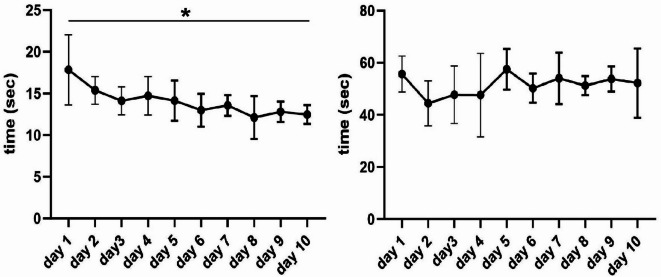
Time required for the pre-intervention safety checklist (left) and dialogic team time-out (right) on 10 consecutive regular working days: With increasing routine, significantly less time was necessary for the examination of the pre-intervention safety-checklist and non-significantly less time was necessary for the examination of the dialogic team-time-out; * *p* < 0.001.

An analysis of the required time for a work-up of the checklist (Fig. 4) showed that after 10 days, an average of 5.4 s significantly less time was necessary in comparison to day 1 (95%-CI: 3.50–7.21, *p* < 0.001) and a maximum of 24.3 s in total. The maximum time needed for the dialogic team-time-out was 67.0 s and mean values also decreased with increasing routine (mean ± s.d.: 55.7 ± 6.9 s vs. 52.2 ± 13.3 s, *p* = 0.610).

## Discussion

The current situation in healthcare is rapidly changing, not only regarding economics, but also and in particular advances in therapy. In order to cope with the demands on interventions, team and patients, we need to get back to basics. These include establishing and maintaining clear communication, effective workflow and good teamwork on the challenges faced daily. Various studies have shown that CRM-based training has a positive long-term impact on employee behaviour, and tools such as checklists can also be effective in healthcare settings^4,13,24^. The WHO Surgical Safety checklist already proved that CRM and TEM can have significant effects on important clinical outcomes in surgery^14,15^. A significant improvement in team communication and teamwork was also observed, which was one of the main objectives of the WHO when introducing the checklist^14^. It therefore seems possible that improving team communication and teamwork could also play a central role in patient safety in endoscopy, especially as current and future interventions in gastrointestinal endoscopy are increasing in terms of their invasiveness and complexity. The present study showed that the perception of team communication and teamwork improved with the introduction of CRM and TEM-based measures based on an individualized approach in direct collaboration with civil aviation experts. These findings are consistent with previous studies which evaluated effects of the introduction of a standard safety checklist in endoscopy^16,18,19,21^. Together with our individually adapted safety checklist and TEM-based dialogic team-time-out the addition of a standardized communication guideline may have promoted clearer and more open communication within the team and thus be a reason for the occurrence of significantly fewer misunderstandings. This is important as it is well known that errors in communication can lead to delay of procedure, inefficiency and tension between team members and therefore to adverse events and a worse patient outcome^25–27^.

Although communication between physicians and nursing assistants was already at a high level on both sides at baseline, the nursing assistants felt significantly better informed about the upcoming procedure by the physician after the CRM implementation. This confirms trends from a previous study in which a non-significant improvement in cooperation between physicians and nurses was reported^17^. Similar to aviation, with different crew composition for almost every flight, there are often constantly changing team constellations in endoscopy, which makes a uniform level of knowledge before the procedure even more essential for safe endoscopy, just as it is crucial in aviation for a safe flight. Because the most important information about the upcoming procedure is checked off within the team using the safety checklist and the dialogic team-time-out, the CRM measures could have played a major role in the improved availability of information for all team members and better communication in general. Both professional groups also stated that they were able to express their concerns more openly to the physicians during the intervention, which could be explained by an increased assertiveness due to the CRM measures.

Another important result of our study was that after the introduction of the CRM measures, a significantly better distribution of tasks around the intervention was perceived. This finding and the tendency towards better resource availability and utilization could be an indication that elementary objectives of CRM may have been achieved through implementation. In times of a shortage of skilled workers and increasing economic pressure, a more effective workflow through CRM could provide comprehensive relief^28^.

The additional questions in the follow-up survey found that most employees did not perceive any delay in the operational process, as delays are widely seen as a potential barrier to the successful introduction of CRM measures^18,19,22,29^. This is supported by our findings that not much additional time is needed to complete the checklist and that the time required becomes significantly shorter with increasing routine. The involvement of employees in the development process could be a reason why a higher proportion of employees felt that communication and teamwork were improved by the individual measures than in previous studies^18,19^. In contrast to the monologic team-time-out, our dialogic team-time-out may have been a difference that contributed to better communication within the team and better teamwork by improving information sharing and collaboration between professional groups. Although our study did not investigate whether the implementations affected patient outcomes, over 80% of staff believed that each of the measures improved patient safety. This belief, together with the finding that almost all staff endorsed the use of a checklist in a potential endoscopic examination of their own, are seen as important factors for sustained and effective use of checklists in the endoscopy unit^22^.

## Strengths and limitations

One of the strengths of our study was that it was conducted in direct cooperation with experts from civil aviation, an area where CRM and TEM measures are standard practice. Although the introduction of checklists in endoscopy has already been the subject of studies, to the best of our knowledge this is the first study worldwide where CRM and TEM measures were implemented individually for the endoscopy unit in direct cooperation with aviation experts. In addition, not only to the expertise from aviation, but also the opinions of the affected employees were included in the development of the CRM and TEM measures right from the start so that they could be optimally adapted to the endoscopy unit. This prevented the inclusion of irrelevant items, which Raphael et al. see as a barrier to the successful implementation of team-time-out^30^. Another strength was the high response rate within the endoscopy department in both surveys, enabling us to get a complete picture of the entire department.

Besides the mentioned strengths the study reveals some limitations. One of such is that the employees helped to develop the measures and evaluated the effect of the measures, which could possibly have led to social desirability bias. The use of external evaluation methods such as structured observations or third-party assessments could help to validate the findings more objectively instead of using subjective outcome measures. In future studies a longitudinal study design with additional follow-up surveys after e.g. 6 and 12 months could help to evaluate the sustainability of behavioural changes in the endoscopy unit. Also, no comparative data were available as the individual approach of transferring aviation-based CRM in direct cooperation with civil aviation experts is a novelty in endoscopy. This, together with the conduction as a single-centre study could lead to the fact that transferability of the results may be impaired. Future studies are mandatory to evaluate the impact of CRM on patient outcomes in prospective multicentre settings and to obtain generalizable results for implementation in all endoscopy units.

## Conclusion

CRM-based interventions individually adapted from civil aviation have the potential to optimize team communication, workflow and teamwork in endoscopy. CRM and TEM had a significant impact on misunderstandings, communication and workflow in endoscopy. A TEM-based dialogic team-time-out showed potential for active integration of all professionals involved in the endoscopic procedures. As this approach is a novelty, it still remains unclear to what extent the results from the study actually affect patient outcomes. Therefore, further studies are needed to provide a clearer picture.

## Supplementary Information

Below is the link to the electronic supplementary material.

Supplementary Material 1

## Data Availability

The datasets used during the current study are available from the corresponding author on reasonable request.

## Abbreviations

CI: Confidence interval
CRM: Crew resource management
IQR: interquartile range
NASA: National Aeronautics and Space Administration
SD: Standard deviation
TEM: Threat and error management
WHO: World Health Organization

## References

[1] Helmreich, R. L., Merritt, A. C. & Wilhelm, J. A. The evolution of crew resource management training in commercial aviation. *Int. J. Aviat. Psychol.* **9**, 19–32 (1999). 11541445 10.1207/s15327108ijap0901_2

[2] Gross, B. et al. Crew resource management training in healthcare: a systematic review of intervention design, training conditions and evaluation. *BMJ Open.* **9**, e025247 (2019). 30826798 10.1136/bmjopen-2018-025247 PMC6410092

[3] Kersten, C., Fink, K., Michels, G. & Busch, H. J. Crew resource management Im schockraum. *Med. Klin. Intensivmed Notfmed*. **116**, 377–388 (2021). 33830287 10.1007/s00063-021-00808-1

[4] O’Dea, A., O’Connor, P. & Keogh, I. A meta-analysis of the effectiveness of crew resource management training in acute care domains. *Postgrad. Med. J.* **90**, 699–708 (2014). 25370080 10.1136/postgradmedj-2014-132800

[5] Buljac-Samardzic, M., Doekhie, K. D. & Van Wijngaarden, J. D. H. Interventions to improve team effectiveness within health care: a systematic review of the past decade. *Human Resourc. Health*. 10.1186/s12960-019-0411-3 (2020). 10.1186/s12960-019-0411-3 PMC6950792 31915007

[6] Brennan, P. A. et al. Review: Avoid, trap, and mitigate – an overview of threat and error management. *Br. J. Oral Maxillofac. Surg.* **58**, 146–150 (2020). 31983481 10.1016/j.bjoms.2020.01.009

[7] Ruskin, K. J. et al. Threat and error management for anesthesiologists. *Curr. Opin. Anaesthesiol.* **26**, 707–713 (2013). 24113268 10.1097/ACO.0000000000000014 PMC4301728

[8] Smith-Jentsch, K. A. et al. Training team performance‐related assertiveness. *Pers. Psychol.* **49**, 909–936 (1996).

[9] Gutgeld-Dror, M., Laor, N. & Karnieli‐Miller, O. Assertiveness in physicians’ interpersonal professional encounters: a scoping review. *Med. Educ.* **58**, 392–404 (2024). 37725417 10.1111/medu.15222

[10] Burke, C. S. How to turn a team of experts into an expert medical team: guidance from the aviation and military communities. *Qual. Saf. Health Care*. **13**, i96–i104 (2004). 15465963 10.1136/qshc.2004.009829 PMC1765796

[11] Salik, I. & Ashurst, J. V. *Closed Loop Communication Training in Medical Simulation* (2025). 31751089

[12] Cooper, S. & Wakelam, A. Leadership of resuscitation teams: ‘Lighthouse leadership’. *Resuscitation* **42**, 27–45 (1999). 10524729 10.1016/s0300-9572(99)00080-5

[13] Clay-Williams, R. & Colligan, L. Back to basics: checklists in aviation and healthcare. *BMJ Qual. Saf.* **24**, 428–431 (2015). 25969512 10.1136/bmjqs-2015-003957 PMC4484042

[14] Sotto, K. T., Burian, B. K. & Brindle, M. E. Impact of the WHO surgical safety checklist relative to its design and intended use: a systematic review and Meta-Meta-Analysis. *J. Am. Coll. Surg.* **233** (809e), 794 (2021). 34592406 10.1016/j.jamcollsurg.2021.08.692

[15] Haynes, A. B. et al. A surgical safety checklist to reduce morbidity and mortality in a global population. *N. Engl. J. Med.* **360**, 491–499 (2009). 19144931 10.1056/NEJMsa0810119

[16] Hefner, J. L. et al. Cultural transformation after implementation of crew resource management: is it really possible? *Am. J. Med. Qual.* **32**, 384–390 (2017). 27422314 10.1177/1062860616655424

[17] Dubois, H., Schmidt, P. T., Creutzfeldt, J. & Bergenmar, M. Person-centered endoscopy safety checklist: Development, implementation, and evaluation. *World J. Gastroenterol.* **23**, 8605–8614 (2017). 29358869 10.3748/wjg.v23.i48.8605 PMC5752721

[18] Kherad, O., Restellini, S., Ménard, C., Martel, M. & Barkun, A. Implementation of a checklist before colonoscopy: a quality improvement initiative. *Endoscopy* **50**, 203–210 (2018). 29237201 10.1055/s-0043-121218

[19] Cherciu Harbiyeli, I. F., Burtea, D. E., Serbanescu, M. S., Nicolau, C. D. & Saftoiu, A. Implementation of a customized safety checklist in Gastrointestinal endoscopy and the importance of team time Out—A Dual-Center pilot study. *Med. (B Aires)*. **59**, 1160 (2023). 10.3390/medicina59061160 PMC10302307 37374363

[20] Wittren, S. P., Gregor, C., Niesen, C. R. & S. R. & Using performance management to implement a preprocedural checklist for Gastrointestinal endoscopy procedures. *Gastroenterol. Nurs.* **42**, 79–83 (2019). 30585912 10.1097/SGA.0000000000000353

[21] Chen, L., Shao, Q., Fang, L., Wei, W. & Jin, J. Construction and application research of a perioperative inspection checklist for acute upper Gastrointestinal bleeding. *J. PeriAnesthesia Nurs.*10.1016/j.jopan.2024.11.002 (2025). 10.1016/j.jopan.2024.11.002 40072393

[22] Gralnek, I. M. et al. Guidance for the implementation of a safety checklist for Gastrointestinal endoscopic procedures: European society of Gastrointestinal endoscopy (ESGE) and European society of gastroenterology and endoscopy nurses and associates (ESGENA) position statement. *Endoscopy* **54**, 206–210 (2022). 34905797 10.1055/a-1695-3244

[23] Borg, I. *Mitarbeiterbefragungen in Der Praxis* (Hogrefe Verlag GMBH & Co.KG, 2015).

[24] Thomassen, Ø., Storesund, A., Søfteland, E. & Brattebø, G. The effects of safety checklists in medicine: a systematic review. *Acta Anaesthesiol. Scand.* **58**, 5–18 (2014). 24116973 10.1111/aas.12207

[25] Lingard, L. Communication failures in the operating room: an observational classification of recurrent types and effects. *Qual. Saf. Health Care*. **13**, 330–334 (2004). 15465935 10.1136/qshc.2003.008425 PMC1743897

[26] The Joint Commission. Sentinel Event Statistics Data: Root Causes by Event Type (2004–2014) (2013). https://Www.Medleague.Com/Wp-Content/Uploads/2013/11/Root_Causes_by_Event_Type_2004-2Q2013.pdf.

[27] Mazzocco, K. et al. Surgical team behaviors and patient outcomes. *Am. J. Surg.* **197**, 678–685 (2009). 18789425 10.1016/j.amjsurg.2008.03.002

[28] Eva Dettmann, D. F. S. M. G. N. V. S. U. L. B. S. Fehlende Fachkräfte in Deutschland – Unterschiede in Den Betrieben Und Mögliche Erklärungsfaktoren: Ergebnisse Aus Dem IAB-Betriebspanel 2018 (2024). https://Doku.Iab.de/Forschungsbericht/2019/Fb1019.pdf.

[29] Bitar, V., Martel, M., Restellini, S., Barkun, A. & Kherad, O. Checklist feasibility and impact in Gastrointestinal endoscopy: a systematic review and narrative synthesis. *Endosc Int. Open.* **09**, E453–E460 (2021). 10.1055/a-1336-3464 PMC7895652 33655049

[30] Raphael, K. et al. Improving patient safety in the endoscopy unit: utilization of remote video auditing to improve time-out compliance. *Gastrointest. Endosc*. **90**, 424–429 (2019). 31054910 10.1016/j.gie.2019.04.237

