# Supplementary material for: Crew resource management and threat and error management improve team communication in endoscopy: a prospective study
Source: Sci Rep. 2025 Nov 3;15:38288. doi: 10.1038/s41598-025-21475-8 (PMC12583798; doi:10.1038/s41598-025-21475-8)
Supplement: Supplementary file 1 — Supplementary Material 1 [file 41598_2025_21475_MOESM1_ESM.docx]

**Supplementary material**

- **A: Standardized communication guideline (excerpt)**

| Clip | | |
| --- | --- | --- |
| Endoscopist |  | Nursing assistant |
| “Clip open” | The clip is opened. | “Clip is open” |
| “Rotate clip” | The clip is rotated to the desired position” | “Rotate clip” |
| “Clip closed” | The clip is closed. | “Clip is closed” |
| “Clip off” | The clip is separated from the insertion aid. | “Clip is off” |
| “Remove clip” | The clip is removed from the working channel. | “Clip is gone” |

- **B: Staff questionnaire:**

| **For both professional groups:** |
| --- |
| 1. Please indicate your gender: |
| 2. What age range are you in? |
| 3. I like working at the Endoscopy Ulm. |
| 4. There is a feeling of security and trust among the staff in our endoscopy unit. |
| 5. I feel encouraged in the endoscopy unit to contribute new ideas or suggestions for improvement. |
| 6. I feel that I and my work are valued within the endoscopy unit. |
| 7. I feel stressed at work. |
| 8. The quality of my work suffers from this stress. |
| 9. Exhaustion affects the quality of my work. |
| 10. If I do not feel fit, I can communicate this openly and refuse to carry out an examination. |
| 11. The safety of our patients is the highest priority on our endoscopy unit; all other issues are subordinate to this. |
| 12. In my opinion, the resources available in the endoscopy unit (staff, materials, information, etc.) are used optimally to make the procedures as safe and effective as possible. |
| 13. All resources (staff, materials, information, etc.) are available to me in our endoscopy unit in order to proceed as safely as possible in the event of complications. |
| 14. Conflicts within the endoscopy unit are addressed openly and dealt with constructively. |
| 15. I can openly communicate mistakes I have made without having to expect negative consequences. |
| 18. We have an atmosphere of trust. I can also communicate constructive criticism and praise to my colleagues across the hierarchy. |
| 19. I regularly receive constructive feedback about my work. |
| 20. I would like to receive more constructive feedback from my colleagues about my work/performance. |
| 21. I regularly give my team members constructive feedback on their work. |
| 22. Before/during/after an intervention, there is a clearly defined distribution of tasks for all members of the treating team. |
| 23. During a procedure, I am always aware of what I can expect from which member of my team and when. |
| 24. The work processes of the two professional groups involved (endoscopists/nursing staff) before/during/after a procedure are well coordinated. |
| 25. If a complication (such as severe bleeding, perforation) arises during a procedure, the treating team is always able to switch quickly from routine to “special case”. |
| 26. In the event of complications, there is a clear allocation of tasks for all team members so that everyone is immediately aware of their role. The situation can therefore be resolved quickly, efficiently and as safely as possible. |
| 27. After a colleague hands over a patient, I often lack important information about the patient. |
| 28. If a team member is relieved during a procedure, all relevant information is communicated to the colleague taking over. |
| 29. All relevant information about the patient is available to me in a clearly structured and complete form before a procedure so that I can quickly gain an overview. |
| 30. Misunderstandings occur during procedures due to unclear communication. |
| 31. Relevant errors concerning the patient or the operating procedure (e.g. adverse event, patient endangerment) occur during interventions due to unclear communication. |
|  |
| **Special questions for physicians:** |
| 16. If I would like the support of another endoscopist during a procedure, I can communicate this openly. |
| 17. If I have concerns during a procedure, I express them openly, even if the concerns relate to the actions of an experienced endoscopist. |
| 32. I attach great importance to a detailed physician-patient consultation before the examination in order to obtain important information for me and to answer all the patient's questions. |
| 33. How much time do you need on average before a procedure to have an adequate doctor-patient consultation? |
| 34. Before we start the procedure, I make sure that all team members are at the same level of knowledge regarding the patient, the procedure, the necessary materials, etc. |
| 35. I find the communication with the nursing assistant during a procedure to be clear and well structured. |
| 36. I find the communication during the procedure with a second endoscopist involved (if present) to be clear and well structured. |
|  |
| **Special questions for nursing assistants:** |
| 16. I would like more support from the physicians during a procedure. |
| 17. If I have any concerns during a procedure, I express them openly, if necessary to an experienced endoscopist. |
| 32. Before we start the procedure, I am informed by the endoscopist about all the necessary information regarding the patient, the procedure, the necessary materials, etc. |
| 33. I find the communication with the endoscopists during a procedure to be clear and well structured. |
| 34. I find the communication during the procedure with a second nursing assistant (if present) to be clear and well structured. |
|  |
| **Questions addressing the CRM-measures:** |
| 1. In my opinion, the introduction of the safety checklist and the team time-out has unnecessarily delayed the investigation process. |
| 2. In my opinion, the safety checklist has... |
| 2 A) ...improved team communication and teamwork in endoscopy. |
| 2 B) ...improved patient safety. |
| 3. In my opinion, the team time-out has... |
| 3 A) ...improved team communication and teamwork in endoscopy. |
| 3 B) ...improved patient safety. |
| 4. In my opinion, the communication guideline has... |
| 4 A) ...improved team communication and teamwork in endoscopy. |
| 4 B) ...improved patient safety. |
| 5. I would advocate the use of a safety checklist for an endoscopic examination performed on me. |
| 6. I have encouraged my colleagues to carry out the safety checklist and the team time-out correctly. |
| 7. If I had any questions or uncertainties, I was able to contact employees or managers without any problems. |
